# Supplementary material for: Chromothripsis during telomere crisis is independent of NHEJ, and consistent with a replicative origin
Source: Genome Res. 2019 May;29(5):737–49. doi: 10.1101/gr.240705.118 (PMC6499312; doi:10.1101/gr.240705.118)
Supplement: Supplemental Material [file supp_gr.240705.118_Supplemental_file_1.zip › contigs/annotated_contigs/DB53/contig.2.DB53_length_412_mean_cov_39.8422330097.docx]

**DB53_length_412_mean_cov_39.8422330097**

```
CCGCTTTCGCCTGCCTTCTTACGGGCCGCGTGTCCTGGGCCAGGGCCGAGATTCCCGTTGCTGCTGCCTCAGCTGGCTTGACCTCTCAT
 >chr4:191002196-191002566 - E=5e-136
TCTGAAACCGAATTTGGACAGTGGGCTCCGGAATGCTGATGGCCTGGGTCAGCCGTTCTCTGGTGGCGATGCCCGGGTATGGGTTCTGC

TCAAAGCAGGCTCGCAGGGCCTAGCTTTGGCTCAGGGTCCAAACGAGTCTCCTTCGCCGTCCCCGTCCCTGGGCTTCCGCGGGGAGGGT

GCCGTCCGAAGGTGTCGGGAGGGCCATCGCGGGGAGCCCCGGCCGGAATTTCACGGACGGACACGGGCAGAGAGAGGCCGGCGGGCTCC

CGTGCACCTCAGCC|AGCGCAA|CCAGGAGGCCTGCCCGGACAGCCAGCCAGCGGCTC                      >chrUn_gl000216:138767-138802 - E=
```
